# DNA damage levels in peripheral blood mononuclear cells before and after first cycle of chemotherapy have comparable prognostic values in germ cell tumor patients

**DOI:** 10.3389/fonc.2024.1360678

**Published:** 2024-03-01

**Authors:** Danica Ivovič, Zuzana Šestáková, Jan Roška, Katarína Kálavská, Lenka Hurbanová, Andrea Holíčková, Božena Smolková, Pavlína Kabelíková, Věra Novotná, Michal Chovanec, Patrik Palacka, Michal Mego, Dana Jurkovičová, Miroslav Chovanec

**Affiliations:** ^1^ Department of Genetics, Cancer Research Institute, Biomedical Research Center, Bratislava, Slovakia; ^2^ Department of Molecular Oncology, Cancer Research Institute, Biomedical Research Center, Bratislava, Slovakia; ^3^ 2^nd^ Department of Oncology, Faculty of Medicine, Comenius University and National Cancer Institute, Bratislava, Slovakia; ^4^ 1^st^ Department of Oncology, St. Elisabeth Cancer Institute, Bratislava, Slovakia

**Keywords:** germ cell tumors, DNA damage level, chemotherapy, prognosis, IGCCCG risk groups, survival, comet assay, peripheral blood mononuclear cells

## Abstract

**Background:**

Germ cell tumors (GCTs) represent the most frequent solid malignancy in young men. This malignancy is highly curable by cisplatin (CDDP)-based chemotherapy. However, there is a proportion of patients having a poor prognosis due to refractory disease or its relapse. No reliable biomarkers being able to timely and accurately stratify poor prognosis GCT patients are currently available. Previously, we have shown that chemotherapy-naïve GCT patients with higher DNA damage levels in peripheral blood mononuclear cells (PBMCs) have significantly worse prognosis compared to patients with lower DNA damage levels.

**Methods:**

DNA damage levels in PBMCs of both chemotherapy-naïve and first cycle chemotherapy-treated GCT patients have been assessed by standard alkaline comet assay and its styrene oxide (SO)-modified version. These levels were correlated with clinico-pathological characteristics.

**Results:**

We re-confirm prognostic value of DNA damage level in chemotherapy-naïve GCT patients and reveal that this prognosticator is equally effective in GCT patients after first cycle of CDDP-based chemotherapy. Furthermore, we demonstrate that SO-modified comet assay is comparably sensitive as standard alkaline comet assay in case of patients who underwent first cycle of CDDP-based chemotherapy, although it appears more suitable to detect DNA cross-links.

**Conclusion:**

We propose that DNA damage levels in PBMCs before and after first cycle of CCDP-based chemotherapy are comparable independent prognosticators for progression-free and overall survivals in GCT patients. Therefore, their clinical use is highly advised to stratify GCT patients to identify those who are most at risk of developing disease recurrence or relapse, allowing tailoring therapeutic interventions to poor prognosis individuals, and optimizing their care management and treatment regimen.

## Introduction

Testicular cancer is the most frequent solid malignancy in young men, but quite rare at the same time in an overall manner, accounting for only around 1% of all cancers. In this malignancy, germ cell tumors (GCTs) represent over 95% of all cases (1). Based on spread, testicular cancer is categorized into the stages 0 - III. Stage 0 (also known as germ cell neoplasia *in situ*) is the earliest stage with the best prognosis for full recovery (2). Risk group classification in metastatic disease is based on the International Germ Cell Cancer Collaborative Group (IGCCCG) criteria defines prognostic factors (histology and location of the primary tumor, location of metastases, and pre-chemotherapy serum marker levels) and categorizes metastatic patients into good, intermediate, and poor prognosis groups (3). The classification system acknowledges two major histologic groups, seminomas and non-seminomas (embryonal carcinoma, yolk sac tumor, choriocarcinoma and teratoma). Non-seminomas can be pure or mixed, and generally are deemed more dangerous since they grow and spread much faster than seminomas (4, 5).

Before the addition of cisplatin (CDDP) to the combination of vinblastine and bleomycine, testicular cancer was, for the patients with advanced disease, usually fatal. After approval by the Food and Drug Administration, CDDP-based chemotherapy is the leading treatment regime for GCT, with survival rate higher than 80%. CDDP is a highly reactive molecule that can bind to a variety of biomolecules, including DNA bases, resulting in the formation of DNA adducts (6). The primary targets in DNA are single nucleophilic N7 sites of purine bases. In addition, reaction of CDDP with DNA may cause covalent linkage of two purines, leading to formation of intrastrand (IaCLs) or interstrand (ICLs) cross-links. CDDP-induced DNA damage can block DNA replication and transcription, which sets off various intracellular signal cascades in the pursue of eliminating these lesions (7, 8).

Despite its high efficiency, CDDP-based therapy fails in some particular GCT cases, leading to poor prognosis, as a consequence of disease relapse or refractory disease. At the cellular level, this can be caused by many different mechanisms, primarily by the altered number of transport proteins, increased drug inactivation, evasion of apoptosis, and altered/aberrant DNA damage response and repair (9, 10). In support of the latter, CDDP-sensitive GCTs have been shown to display a deficiency in DNA repair mechanisms and exhibit a decreased ability to remove CDDP-induced DNA lesions (11). In addition, lower levels of some of the key players of nucleotide excision repair (NER), namely XPA, ERCC1 and XPF, were found in GCTs (12–14).

Even though the benefits of CDDP-based chemotherapy exceed long-term risks (15), a further optimization of management of GCT patients is still necessary. In this malignancy, there is a need to seek: on the one hand for more effective treatment options in case of poor prognosis patients, and for avoiding the toxic effects of needlessly applied chemotherapy in good prognosis patients on the other. Therefore, searching for novel, more effective prognostic criteria/markers is crucially required. Previously, we have shown that chemotherapy-naïve GCT patients with higher DNA damage levels in peripheral blood mononuclear cells (PBMCs) have significantly worse progression-free and overall survival (PFS and OS, respectively) compared to patients with lower DNA damage levels. In addition, we have revealed the added prognostic value of DNA damage level in combination with the IGCCCG risk groups. Therefore, DNA damage level was suggested to be an independent prognosticator for PFS and OS in chemotherapy-naïve GCT patients and its clinical use, particularly in combination with the IGCCCG risk groups, may help in stratifying these patients (16, 17).

As mentioned above, cytotoxicity of CDDP is manifested through DNA cross-links, mainly ICLs, which cannot be readily detected with the standard alkaline comet assay. Therefore, modified versions of this assay have been developed to detect ICLs, particularly for the purpose of monitoring of patients’ sensitivity and chemotherapy response. In these modifications, a fixed level of random DNA single-strand breakage is delivered into cells’ DNA, leading to its unwinding under alkaline conditions. This allows that ICLs, DNA damage type being responsible for the majority of DNA retention in the comet head observed in the standard alkaline comet assay, became detectable through decrease in % DNA in tail after treatment with DNA single-strand breaks (SSBs)-inducing agents, such as gamma rays (18–20), methyl methanesulfonate (21), hydrogen peroxide (22), and styrene oxide (SO) (23). In the present study, SO was chosen as an SSBs-inducing agent (24) enabling ICL detection and both SO-modified and standard alkaline comet assays were used in parallel to find out whether difference between the DNA damage levels before and after first cycle of CDDP-based chemotherapy could even more accurately stratify GCT patients with regard to their prognosis, as we hypothesized that the difference between these two DNA damage levels should be more relevant to prognosis, because it reflects the DNA damage level caused by chemotherapy *per se* and such DNA damage should translate into the cytotoxic effects.

## Materials and methods

### Subjects

The present study involved 132 GCT patients treated from January 2013 to November 2019 in the National Cancer Institute and/or the St. Elisabeth Cancer Institute, Bratislava, Slovakia (**Table 1**). Patients with concurrent malignancy other than non-melanoma skin cancer in the previous 5 years were excluded from the study. Clinical stage of the disease was assigned according to the 2016 American Joint Committee on Cancer staging manual (25). Data regarding age, tumor histologic subtype, clinical stage and type and number of metastatic lesions were documented for all patients. The control group was composed of 11 healthy, age-matched (age range 28-60 y, median 35 y) male volunteers, who were not occupationally exposed to genotoxic chemicals. Age did not differ significantly between cases and controls (*p* = 0.289). No chronic disease was reported in the control group. Levels of DNA damage in controls served only for the comparison of patients with a healthy population, and thereby provided quality control of experimental approaches used in our study.

**Table 1:** Patient characteristics.

Variable	N	%
**All patients**	132	100
Histology		
Seminoma	41	31
Non-seminoma	91	69
IGCCCG risk group^*^		
Good prognosis	81	65
Intermediate prognosis	32	26
Poor prognosis	12	9
Sites of metastases		
Retroperitoneum	107	81
Mediastinum	16	12
Lungs	31	24
Liver	13	10
Brain	6	5
Other	10	8
No. of metastatic sites		
0	18	14
1 to 2	88	66
≥ 3	26	20
Staging (UICG)		
IA	1	0.5
IB	6	4
IS	10	7.5
IIA	16	12
IIB	26	20
IIC	17	13
IIIA	12	9
IIIB	20	15
IIIC	24	19
Response to therapy		
Favourable response	108	82
Unfavourable response	24	18

^*^1 and 6 patients were of IA and IB stage respectively, and therefore were not included in IGCCCG risk group classification.

IGCCCG, International Germ Cell Consensus Classification Group; UICC, Union for International Cancer Control.

The present study (protocol IZLO1, Chair: M. Mego) was approved by the Institutional Review Board and Ethical committee of the National Cancer Institute, Bratislava, Slovakia. All participants were recruited and consented according to the approved protocol. They all signed informed consent before study enrolment.

### Peripheral blood mononuclear cells preparation

Peripheral blood was collected into lithium-heparin treated tubes (BD, Vacutainer Blood Collection Tubes) at baseline in the morning on day -3 to 0 of the first cycle of chemotherapy and on day 6 to 43 (median 21) after chemotherapy. Peripheral blood was diluted 1:1 with phosphate-buffered saline (PBS; 137 mM NaCl, 8 mM Na_2_HPO_4_, 2.7 mM KCl, 1.8 mM KH_2_PO_4_, pH 7.2) and the resulting mixture was carefully poured onto Histopaque-1077 (Sigma-Aldrich, Germany). After centrifugation at mild conditions, leading to blood cells layering and PBMCs separation, PBMC pellets were washed twice with, and resuspended in, PBS. Afterwards, PMBC pellets were cryopreserved. For cryopreservation, they were resuspended in freezing medium (10% DMSO, 40% RPMI 1640 cell culture medium, 50% foetal bovine serum) at a density of 1 × 10^6^ cells/mL and slowly deep-frozen in a Nalgene “Mr. Frosty” freezing container to -80°C within 24 h. Subsequently, frozen PMBCs were transferred to, and stored in, liquid nitrogen until used.

### Standard alkaline comet assay

The standard comet assay was carried out under alkaline conditions, as previously described (16, 17, 26). Microscope slides were pre-coated with 1% normal melting point agarose (NMP, Sigma). PBMCs were thawed at 37°C, washed with PBS, and 8 × 10^4^ cells were mixed with low melting point agarose (LMP, Sigma) at a final concentration of 0.75%. Cell mixtures were spread onto frosted microscopic slides covered with NMP and then covered with coverslips to make a uniform layer over the NMP agarose. Slides were kept at 4°C until the agarose solidified. After removal of the coverslip, the samples were put into PBS. After 30 min, the cells were lysed in cold lysis buffer (2.5 M NaCl, 10 mM Tris-HCl, 100 mM Na_2_EDTA, pH 10.0) containing 1% Triton-X for 60 min at 4°C. After lysis, slides were arranged in electrophoresis tank filled with cold electrophoresis buffer (1 mM Na_2_EDTA, 0.3 M NaOH, pH 13.0) for 30 min at 4°C. Electrophoresis was performed at 0.7 V/cm, 300 mA for 30 min at 4°C. Following electrophoresis, slides were neutralized in 0.4 M Tris-HCl, pH 7.5. The slides were then washed with distilled H_2_O, fixed in alcohol and let dry overnight at room temperature (RT). Each slide was stained with ethidium bromide (30 μg/mL, 20 min at RT). 100 randomly selected nucleoids *per* slide (300 nucleoids for every patient sample) were analysed through the Metafer-MetaCyte analysing software (Metasystems, Altlussheim, Germany), and the DNA damage was expressed as mean % DNA in tail ± standard error of the mean (SEM). DNA damage in chemotherapy-naïve GCT patients did not differ significantly from that of healthy controls (6.34% ± 0.32 *vs*. 6.85% ± 0.88, *p* = 0.102).

### SO-modified comet assay

To detect CDDP-induced DNA cross-links, particularly ICLs, SO-modified alkaline comet assay was used. The methodology is the same as described above with the exception that upon solidification of agarose and removal of the coverslip, the samples were immersed in SO at a final concentration of 600 µM. After a 30 min incubation period, cell lysis was carried out using the same cold lysis buffer, and the subsequent steps were conducted in parallel with the standard alkaline comet assay.

### Statistical analysis

The patient characteristics were tabulated and summarized as the median (range) values for continuous variables and frequency (percentage) for categorical variables, respectively. Kolmogorov-Smirnov test was used to assess normality of data distribution. If normally distributed, Student t-test or analysis of variance with the Bonferroni’s or Tamhane’s corrections were used, depending on the homogeneity of variance. Non-parametric Mann-Whitney U or Kruskal-Wallis H-tests were used for non-normally distributed data, whereas the Fisher’s exact test was used when % DNA in tail was categorized according to the cut-off value. Receiver operator characteristic (ROC) analyses coupled with the calculation of the Youden (27) index were applied to determine the optimal cut-off value with the highest sensitivity and specificity of the DNA damage level and to evaluate its prognostic accuracy. The median follow-up period was calculated as the median observation time of all patients, including patients who were alive at the time of the last follow-up. PFS was calculated from the date of starting CDDP-based chemotherapy to the date of progression or death, or date of the last follow-up. OS was calculated from the starting date of systemic therapy to the date of death or last follow-up. PFS and OS were estimated by the Kaplan-Meier product-limit method with log-rank test to determine the differences between survival curves. Estimates of hazard ratio (HR) for the % DNA in tail above the cut-off value for all patients and patients stratified according to the individual clinical categories were calculated using the univariate Cox proportional hazard regression analysis. Due to low number of events in intermediate and poor prognosis IGCCCG risk groups, these groups were combined for multivariate Cox proportional hazard regression analysis, where factors affecting PFS and OS were determined. All presented *p* values were two-tailed, with *p* < 0.05 considered as significant. Statistical analyses were performed using IBM SPSS statistics version 23.0 software for Windows (IBM). The suitability of the statistical approaches used here to analyze and interpret the comet assay data has repeatedly been verified (28, 29).

## Results

### Patients’ characteristics

Study cohort consisted of 132 GCT patients. Worthwhile to mention, there is an overlap in patient samples with our two previous studies, with 58 and 30 patients also appearing in cohorts from 2020 (17) and 2016 (16), respectively. The number of patients appearing in all three cohorts is 29. Basic and clinical patients’ characteristics are summarized in **Table 1**. The median age of patients was 35 y (range 19-62 y). The majority of patients had a good prognosis (75%) according to IGCCCG. The 2-year PFS of patients with good, intermediate and poor prognosis was 92%, 85% and 37%, and the 5-year OS was 95%, 93%, and 64%, respectively. Tumor specimens were represented by 41 pure seminomas (31%), 56 non-seminomas (42%) (22 embryonal carcinomas, 16 yolk sac tumors, 10 choriocarcinomas and 8 teratomas) and 35 mixed GCTs (27%) (17 non-seminoma mixes and 18 non-seminomas mixed with seminomas). 106 patients (80%) were treated with BEP (bleomycin, etoposide, CDDP) regimen, 22 patients (17%) were treated with TIP (paclitaxel, ifosfamid, CDDP) or combination of BEP/TIP (30) and 4 patients (3%) received EP (etoposide, CDDP) chemotherapy. After chemotherapy, all patients were given granulocyte-colony stimulating factor support (filgrastim or pegfilgrastim). 3 seminoma patients (2.3%) underwent previous radiation therapy in adjuvant setting and/or for stage II disease.

### Correlation between DNA damage levels in PBMCs and clinical characteristics in chemotherapy-naïve GCT patients

In our first study (16), the cut-off value was 5.25% ± 0.60 (the mean of DNA damage level in PBMCs of chemotherapy-naïve GCT patients measured by standard alkaline comet assay as % DNA in tail ± SEM). The mean in the enlarged dataset of subsequent study (17) was 5.49% ± 0.32. However, the larger sample size in the latter study allowed us to use ROC curve analysis to calculate the % DNA in tail cut-off value providing the highest sensitivity and specificity for an adverse outcome (disease progression or mortality). Based on the area under curve value 0.813 with standard error (SE) = 0.046 (95% confidence interval [CI]: 0.723-0.902, *p* < 0.001) for disease progression and 0.814 with SE = 0.062 (95% CI: 0.693-0.934, *p* = 0.001) for mortality, the cut-off value was refined to 6.34 (17). Importantly, both cut-off values, even though slightly different, were significantly associated with prognosis, and first cut-off value (16) remained significant in the follow-up study (17). For the present patient cohort, the cut-off value of DNA damage level in PBMCs of chemotherapy-naïve GCT patients for PFS and OS was calculated to be 6.34 and 5.26% DNA in tail, respectively.

### Prognostic value of DNA damage level in PBMCs of chemotherapy-naïve GCT patients

**Figure 1:**
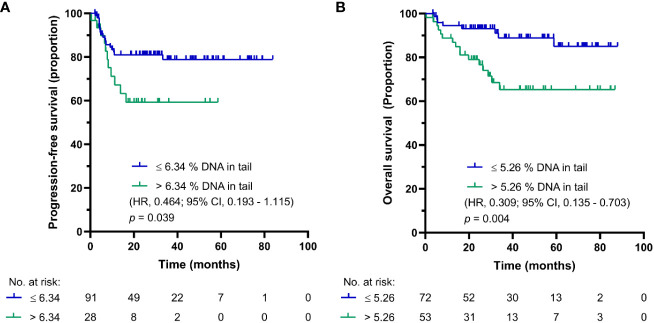
Kaplan-Meier survival curves for DNA damage level measured by standard alkaline comet assay as % DNA in tail in PBMCs of chemotherapy-naïve GCT patients. **(A)** PFS and **(B)** OS.

The median follow-up was 44.2 months (0.4-83.8 months) and the median follow-up for patients being alive at the time of analysis was 53.4 months (0.4-88 months). The prognostic value was evaluated by the Kaplan-Meier method. The Kaplan-Meier estimates for PFS and OS are shown in **Figures 1A, B**, respectively. For chemotherapy-naïve GCT patients with DNA damage levels above the cut-off value, the log-rank test showed significantly reduced PFS (*p* = 0.039) and OS (*p* = 0.004). While chemotherapy-naïve GCT patients with DNA damage levels ≤ 6.34% DNA in tail showed 2-year PFS of 83%, those with DNA damage levels > 6.34% DNA in tail displayed 2-year PFS of 60%. The 5-year estimates of OS were 90% for chemotherapy-naïve GCT patients with % DNA in tail ≤ 5.26 and 64% for those with % DNA in tail > 5.26.

**Figure 2:**
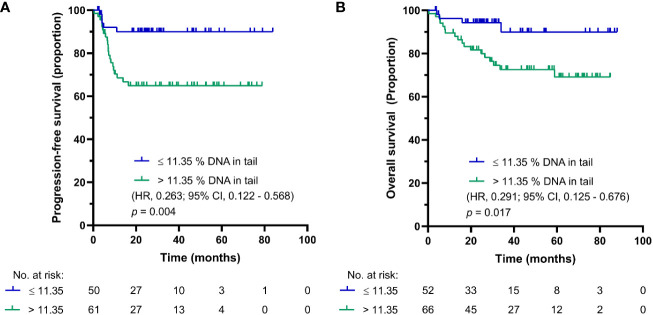
Kaplan-Meier survival curves for DNA damage level measured by SO-modified comet assay as % DNA in tail in PBMCs of chemotherapy-naïve GCT patients. **(A)** PFS and **(B)** OS.

SO-modified comet assay, also called reverse comet assay, was used in parallel in this study. The main reasoning for the inclusion of this form of comet assay was to compare DNA damage levels in PBMCs of GCT patients before and after first cycle chemotherapy, where occurrence of DNA cross-links is expected after CDDP administration. Using this modification, the cut-off value for PFS and OS synchronized into one value of 11.35% DNA in tail in chemotherapy-naïve GCT patients. The Kaplan-Meier estimates for PFS and OS are shown in **Figures 2A, B**, respectively. For patients with DNA damage levels above the cut-off value, the log-rank test again showed significantly reduced PFS (*p* = 0.004) and OS (*p* = 0.017). Patients with DNA damage levels ≤ 11.35% DNA in tail showed 2-year PFS of 95%, and those with DNA damage levels > 11.35% DNA in tail displayed 2-year PFS of 62%. The 5-year estimate of OS was 96% for chemotherapy-naïve GCT patients with % DNA in tail ≤ 11.35 and 67% for those with % DNA in tail > 11.35%.

### Correlation between the DNA damage levels in PBMCs of GCT patients after first cycle chemotherapy and clinical characteristics

**Figure 3:**
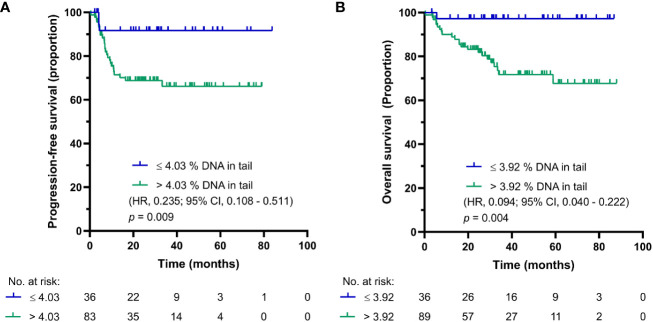
Kaplan-Meier survival curves for DNA damage level measured by standard alkaline comet assay as % DNA in tail in PBMCs of GCT patients after first cycle of CDDP-based chemotherapy. **(A)** PFS and **(B)** OS.

After first cycle of CDDP-based chemotherapy, the cut-off values for PFS and OS were calculated to be 4.025 and 3.9% DNA in tail, respectively. The Kaplan-Meier estimates for PFS and OS are shown in **Figures 3A, B**, respectively. For GCT patients after first cycle of chemotherapy with DNA damage levels above the cut-off value, the log-rank test showed significantly reduced PFS (*p* = 0.009) and OS (*p* = 0.004). While patients after first cycle of chemotherapy with DNA damage levels ≤ 4.025% DNA in tail showed 2-year PFS of 95%, those with DNA damage levels > 4.025% DNA in tail displayed 2-year PFS of 66%. The 5-year estimates of OS were 97% for GCT patients with % DNA in tail ≤ 3.9 and 64% for those with % DNA in tail > 3.9.

**Figure 4:**
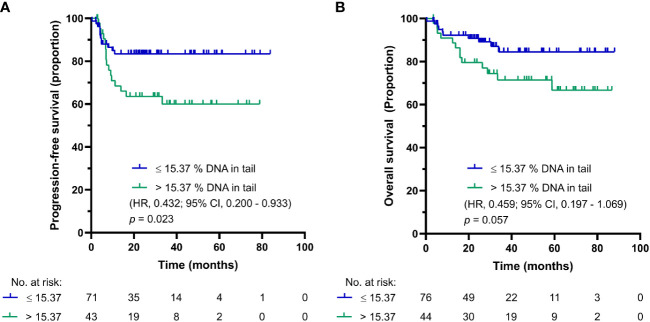
Kaplan-Meier survival curves for DNA damage level measured by SO-modified comet assay as % DNA in tail in PBMCs of GCT patients after first cycle of CDDP-based chemotherapy. **(A)** PFS and **(B)** OS.

As in the case of DNA damage level in PBMCs of chemotherapy-naïve GCT patients, the use of SO-modified comet assay synchronized the cut-off for PFS and OS into one value of 15.37% DNA in tail in GCT patients after first cycle of CDDP-based chemotherapy. The Kaplan-Meier estimates for PFS and OS are shown in **Figures 4A, B**, respectively. For GCT patients after first cycle CDDP-based chemotherapy with DNA damage levels above the cut-off value, the log-rank test showed significantly reduced PFS (*p* = 0.023), but not OS (*p* = 0.057). While patients with DNA damage levels ≤ 15.37% DNA in tail showed 2-year PFS of 85%, those with DNA damage levels > 15.37% DNA in tail displayed PFS of 61%. The 5-year estimate of OS was 83% for GCT patients after first cycle of CDDP-based chemotherapy with % DNA in tail ≤ 15.37 and 72% for those with DNA damage levels > 15.37% DNA in tail.

As obvious (**Table 2**), multivariate Cox regression analysis confirmed prognostic significance of DNA damage level > 5.25 (measured by standard alkaline comet assay) in chemotherapy-naïve GCT patients for OS (HR = 2.96, 95 % CI: 1.23-7.13, *p* = 0.015). In addition, in this analysis, % DNA in tail above the cut-off value of 4.03 (measured by standard alkaline comet assay) increased the risk of having higher risk of disease relapse (determined as shorter PFS) in GCT patients after first cycle of chemotherapy (HR = 3.414, 95 % CI: 1.02-11.38, *p* = 0.046). Finally, % DNA in tail above the cut-off value of 3.92 (measured by standard alkaline comet assay) increased the risk of having shorter OS in GCT patients after first cycle of chemotherapy (HR = 8.84, 95 % CI: 1.19-65.73, *p* = 0.033).

**Table 2:** Hazard risk from multivariate Cox models for PFS and OS.

Variable	PFS	
	HR (95% CI)	*p* value^†^
DNA damage level before chemotherapy > 6.34% DNA in tail	1.68 (0.76 – 3.72)	0.198
IGCCCG risk group^‡^	7.96 (3.33 – 19.02)	**<0.001**
DNA damage level before chemotherapy > 11.35^*^ % DNA in tail	2.41 (0.89 – 6.54)	0.084
IGCCCG risk group	9.91 (3.65 – 26.92)	**<0.001**
DNA damage level after first cycle of chemotherapy > 4.03% DNA in tail	3.41 (1.02 – 11.38)	**0.046**
IGCCCG risk group	7.92 (3.32 – 18.85)	**<0.001**
DNA damage level after first cycle of chemotherapy > 15.37^*^ % DNA in tail	1.87 (0.86 – 4.04)	0.114
IGCCCG risk group	9.39 (3.74 – 23.58)	**<0.001**
Variable	OS	
	HR (95% CI)	*p* value^†^
DNA damage level before chemotherapy > 5.25% DNA in tail	2.96 (1.23 – 7.13)	**0.015**
IGCCCG risk group	9.28 (3.42 – 25.20)	**<0.001**
DNA damage level before chemotherapy > 11.35^*^ % DNA in tail	2.37 (0.79 – 7.07)	0.123
IGCCCG risk group	10.60 (3.53 – 31.81)	**<0.001**
DNA damage level after first cycle of chemotherapy > 3.92% DNA in tail	8.84 (1.19 – 65.73)	**0.033**
IGCCCG risk group	8.86 (3.29 – 23.91)	**<0.001**
DNA damage level after first cycle of chemotherapy > 15.37^*^% DNA in tail	1.92 (0.84 – 4.39)	0.122
IGCCCG risk group	11.40 (3.85 – 33.75)	**<0.001**

^*^DNA damage levels assessed by SO-modified comet assay.

^†^Significant *p* values are in bold.

IGCCCG, International Germ Cell Consensus Classification Group; HR, hazard ratio; CI, confidence interval; PFS, progression-free survival; OS, overall survival. ^‡^ 1 and 6 patients were of IA and IB stage respectively, and therefore were not included in the IGCCCG risk group classification shown in **Table 1**, but are included in the statistical analysis shown herein.

## Discussion

We have previously reported that chemotherapy-naïve GCT patients with higher DNA damage levels in PBMCs have significantly worse prognosis compared to patients with lower DNA damage levels, indicating prognostic value of DNA damage level in this malignity (16, 17). Furthermore, we revealed the added prognostic value of DNA damage level in combination with IGCCCG risk group (17). Collectively, our previous findings suggested clinical use of measurement of DNA damage level in PBMCs (particularly in combination with IGCCCG risk groups) as independent prognosticator for PFS and OS in chemotherapy-naïve GCT patients.

The primary aim of this study was to find out whether difference between the DNA damage levels before and after first cycle of CDDP-based chemotherapy could even more accurately stratify GCT patients with regard to their prognosis. We hypothesized that difference between these two DNA damage levels should be more relevant to prognosis of GCT patients than DNA damage level of chemotherapy-naïve patients, as it represents the level of DNA damage caused by chemotherapy and such DNA damage should translate into anti-cancer cytotoxic effects. Unfortunately, difference in the DNA damage level in PMBCs before and after first cycle of CDDP-based chemotherapy (*i*. *e*. the DNA damage level, which represents subtraction of the DNA damage level before chemotherapy from the DNA damage level after chemotherapy) did not show any statistically significant correlation with prognosis of GCT patients. A plausible explanation for this finding could be that sample collection on day 21 (median value) after chemotherapy administration was a little bit late to detect therapeutic DNA damage levels (the highest DNA damage levels that can be reached by chemotherapy). If samples were taken earlier after chemotherapy administration (*e*. *g*. on day 2 or 3), higher levels of CDDP-induced DNA damage levels and, as a consequence, bigger difference between the DNA damage levels before and after chemotherapy would expectedly be detected, with a possibility of existence of significant correlation of DNA damage level difference with prognosis in such a case. In line with this assumption are findings by Fikrová et al. (23) who demonstrated decrease of % DNA in tail in peripheral lymphocytes of patients with non-small cell lung carcinoma receiving platinum derivative-based chemotherapy immediately and first days after its administration using the alkaline comet assay. Later (after one week), % DNA in tail increased although it did not reach the levels before chemotherapy. Our sample collection model adopted general clinical practice in the country, so if difference between the DNA damage levels before and after chemotherapy is meant to be used as prognosticator, sample collection model would have to be modified accordingly. Nevertheless, these experiments revealed that DNA damage level in PBMCs is as effective prognosticator in GCT patients after first cycle of CDDP-based chemotherapy as in chemotherapy-naïve GCT patients.

To be able to more specifically detect DNA damage induced by CDDP, SO-modified comet assay was used in this study in parallel with standard alkaline comet assay. SO-modified comet assay allows detection of ICLs, critical cytotoxic DNA lesions induced by CDDP that are responsible for retaining most DNA in the head of comet in alkaline comet assay. As evident, and consistent with our hypothesis, SO-modified comet assay detected higher level of DNA damage after first cycle of chemotherapy compared to chemotherapy-naïve DNA damage level in the same individual, providing evidence that CDDP-based chemotherapy indeed induces therapeutically relevant ICLs *in vivo*, which can be easily quantified by subtracting % DNA in tail after and before chemotherapy obtained by SO-modified comet assay. Notably, even standard alkaline comet assay was able to detect ICLs: in contrast to DNA damage levels detected by SO-modified comet assay, DNA damage levels after first cycle of CDDP-based chemotherapy were lower than those before chemotherapy as measured by standard alkaline comet assay, proving that CDDP-induced ICLs indeed retain DNA in comet head under such conditions. Both methods are therefore applicable for the detection of CDDP-induced DNA damage in clinical use, although the SO-modified version is significantly more sensitive as it provides a wider window for DNA damage detection. Another advantage of SO-modified version of the comet assays lies in the unified cut-off value for PFS and OS and higher reproducibility of data obtained, both being strong adjunctive factors in the clinical use of this method to determine the level of DNA damage for prognostic purposes.

Translated into the clinical context, SO-modified comet assay could help more accurately optimize care management and treatment regimen of GCT patients. In case of ambiguity in patient stratification according to prognosis using IGCCCG risk group classification, DNA damage level data obtained by SO-modified comet assay may have the added value to clarify prognosis. If prognosis turns to be good based on this combined approach, GCT patients, especially those of clinical stage I, can potentially avoid chemotherapy and hence its adverse effects, and in case of worse-to-poor prognosis, treatment alternative to standard protocol can be an option to provide such patients with more aggressive treatment to improve their outcome.

Previous studies have found a good correlation between CDDP-DNA adduct level in peripheral blood cells and therapy outcome in various cancers including testicular cancer (31), suggesting that monitoring of CDDP-induced DNA damage level in peripheral blood cells might be informative with regard to patient responsiveness to CDDP. Indeed, in testicular cancer, CDDP-induced DNA damage in peripheral blood cells was shown to clearly correlate with survival, as shown herein, and with the occurrence of complete response in non-seminomatous patients with poor prognosis (32). In addition, studies examining CDDP-induced DNA damage may help to determine relationship between the DNA damage in peripheral blood and internal organ response to exposure to CDDP *in vivo* (31) and to predict the efficacy of the chemotherapy and tailor subsequent patient management. However, there is caveat to using CDDP-induced DNA damage level to predict post-chemotherapy prognosis in flat scale manner, as individual patients may significantly differ by as much as 10^3^ in their CDDP-DNA adduct levels (31). Instead, algorithm counting also chemotherapy-naïve DNA level and combing more prognostic factors is rather advised.

Comparing DNA damage levels before and after chemotherapy using both comet assay methods allowed us to reveal that GCT patients before administration of CDDP-based chemotherapy contain certain level of ICLs in their DNA in PBMCs. This is not surprising because there are numerous endogenously produced compounds in cell that can induce cross-links in DNA. The most well-characterized potential sources of ICLs are the by-products of lipid peroxidation, psoralens, acetaldehyde, and few more (33). Question is whether these DNA cross-links represent factor that had contributed to cancer development in these GCT patients. Unfortunately, we do not have information on ICLs in healthy individuals, only the total DNA damage data, and therefore we are unable to address this issue.

In addition to limitation of our study regarding time of sample collection after chemotherapy, another limitation may relate to histological heterogeneity of GCT. As it seems that non-seminoma displays higher levels of endogenous DNA damage (16, 34) compared to seminoma, potential clinical use of DNA damage level to stratify GCT patients to reveal their prognosis should take into consideration histological subtype of the disease.

## Conclusion

The present study assessed DNA damage levels in PBMCs of both chemotherapy-naïve GCT patients and GCT patients after first cycle of CDDP-based chemotherapy to revise and extend our previous findings. First, we re-confirm prognostic value of DNA damage level in chemotherapy-naïve GCT patients and reveal that this prognosticator is equally effective in patients after first cycle of CDDP-based chemotherapy. Second, we demonstrate that SO-modified comet assay may have better clinical use to measure DNA damage compared to standard alkaline assay, as it provides the same cut-off value for PFS and OS and provides higher reproducibility of data, the two facts representing an important advantage in the clinical use. Moreover, this modification detects a large proportion of CDDP-induced DNA lesions compared to standard alkaline comet assay, providing a wider window for stratification of GCT patients. In summary, DNA damage levels in PBMCs before and after first cycle of CDDP-based chemotherapy are comparable as to their prognostic value in GCTs. SO-modified comet assay, however, detects a wider range of DNA damage types, allowing more precise poor prognosis risk stratification in this malignity.

## Data availability statement

The original contributions presented in the study are included in the article/supplementary material. Further inquiries can be directed to the corresponding author.

## Ethics statement

The studies involving humans were approved by Institutional Review and Ethical Committee of the National Cancer Institute, Bratislava, Slovakia. The studies were conducted in accordance with the local legislation and institutional requirements. The participants provided their written informed consent to participate in this study.

## Author contributions

DI: Conceptualization, Data curation, Investigation, Methodology, Writing – original draft. ZŠ: Conceptualization, Data curation, Investigation, Methodology, Writing – review & editing, Validation. JR: Data curation, Formal analysis, Software, Writing – original draft. KK: Conceptualization, Data curation, Formal analysis, Investigation, Writing – review & editing. LH: Formal analysis, Methodology, Writing – review & editing. AH: Formal analysis, Methodology, Writing – review & editing. BS: Data curation, Formal analysis, Software, Writing – review & editing. PK: Data curation, Investigation, Writing – review & editing. VN: Conceptualization, Formal analysis, Writing – review & editing. MicC: Conceptualization, Data curation, Formal analysis, Writing – review & editing. PP: Conceptualization, Data curation, Formal analysis, Writing – review & editing. MM: Conceptualization, Data curation, Formal analysis, Funding acquisition, Writing – review & editing. DJ: Conceptualization, Funding acquisition, Investigation, Writing – review & editing. MirC: Conceptualization, Data curation, Formal analysis, Funding acquisition, Investigation, Project administration, Resources, Writing – original draft.
